# Obesity in Primary Care: A Comprehensive Approach for Family Physicians

**DOI:** 10.7759/cureus.90742

**Published:** 2025-08-22

**Authors:** Madhavi Medipally, Arnaz Avasia, Mruganka Parasnis

**Affiliations:** 1 Family Medicine, University of Massachusetts, Shrewsbury, USA; 2 Public Health, University of Alabama at Birmingham, Birmingham, USA; 3 Biomedical Engineering, Tufts University, Medford, USA

**Keywords:** chronic disease management, comprehensive review, lifestyle diseases, obesity, primary care

## Abstract

Obesity is a disease driven by the interplay of varied factors such as genetic, environmental, and behavioral factors. It can pose a risk for numerous comorbidities, including type 2 diabetes, cardiovascular disease, obstructive sleep apnea, and cancer. Despite its prevalence and health consequences, obesity remains substantially undertreated in primary care. This review highlights the current understanding of obesity pathophysiology, classification, and epidemiology, with an emphasis on clinical management in the primary care setting. Evidence-based strategies for prevention, screening, and treatment, including lifestyle interventions, behavioral counseling, pharmacotherapy, and surgical options, are being presented. Obesity management requires a patient-centered, multidisciplinary approach. Primary care providers play a pivotal role in initiating care through routine assessment (body mass index (BMI), waist circumference, and neck circumference), addressing different challenges, and delivering counseling with the help of motivational interviewing. Primary care is a critical frontline setting for obesity prevention and treatment. Enhancing provider education, adopting structured counseling models (e.g., 5As: assess, advise, agree, assist, and arrange), leveraging telehealth, and expanding access to multidisciplinary teams can improve outcomes. Addressing obesity proactively and comprehensively in primary care, along with shared decision-making (SDM) where patients and physicians can mutually create a treatment plan, can reduce the burden of chronic disease and improve population health.

## Introduction and background

Obesity is caused by the interaction of multiple factors, such as environmental, genetic, and behavioral. This results in the accumulation of adipose tissue and is associated with reduced quality of life and leads to other comorbidities like diabetes, cardiovascular diseases, and cancer [1]. Obesity is also a chronically undertreated condition, with only 40% of adults overweight or obese receiving counseling to lose weight [2].

Overweight and obesity are defined as excessive fat accumulation that presents a health risk. A body mass index (BMI) over 25 is considered overweight, and that above 30 is considered obese [1]. Overweight is a BMI between 25 and 29.9 kg/m^2^. Obesity is further classified as class I obesity (BMI 30-34.9 kg/m^2^), class II obesity (BMI 35-39.9 kg/m^2^), and class III obesity (BMI ≥ 40 kg/m^2^) [2].

According to the CDC, obesity is a chronic health condition that increases the risk for coronary heart disease, type 2 diabetes, hypertension, stroke, and some cancers [3]. The prevalence of obesity has increased 300% since 1975 in the whole world and is one of the leading causes of death worldwide and in the United States [2].

As per the CDC, the average prevalence of obesity among adults from August 2021 to August 2023 was 40.3%, 39.2% in men and 41.3% in women. The prevalence of obesity was higher (46.4%) in adults aged 40-59 as compared to that in adults aged 20-39 (35.5%) and 60 and older (38.9%) in both men and women. The prevalence of obesity was lower in adults with a college degree or more (31.6%) as compared to adults with lesser education (high school diploma or less, 44.6%) and some college (45.0%) [3].

The global prevalence of obesity has risen, with the average BMI of adults rising from 22 kg/m^2^ in 1975 to 24 kg/m^2^ in 2014. The occurrence of obesity has increased from 3.2% to 10.8% in men and from 6.4% to 14.9% in women [4,5].

Primary care physicians (PCPs) are the first point of contact for most patients with obesity, and they deliver early diagnosis, counseling, treatment, and continued monitoring to the patients. PCPs have a central role in obesity management, even though obesity is a societal problem that requires policy-level interventions and changes in food regulations. There are key barriers to care, such as time constraints and a lack of training regarding nutritional counseling. This research article explores the current understanding of obesity pathophysiology, classification, and epidemiology, with an emphasis on prevention and clinical management in the primary care setting, and aims to make suggestions to improve the current state of obesity management by the PCPs. This article aims to provide evidence-based, patient-centered guidelines for the management of obesity in primary care, including counseling, lifestyle modifications, pharmacotherapy, and surgical options, and also discusses associated barriers and the importance of reducing social stigma associated with obesity as a narrative review.

## Review

Methods

For this research, we searched through the last 15 years of articles from Google Scholar, Web of Science, PubMed, and Scopus and chose the articles mentioning obesity management. The current definition and prevalence of obesity in the United States are taken from the WHO and CDC websites, respectively. One researcher collected the initial collection of material, another researcher reviewed the content, and a third researcher went through the manuscript and made sure the research mentioned in the manuscript was referenced appropriately. We have outlined the following various stages in which PCPs manage their patients regarding obesity and the measures taken by the PCPs to manage the condition.

Obesity management

Obesity management is discussed as prevention, treatment, counseling, lifestyle interventions, pharmacotherapy, surgery, and barriers associated with the treatment of obesity in the following sections.

Prevention in Primary Care

Ideally, it would be optimal for the PCPs to prevent obesity. Obesity primary prevention is crucial, as it is more expensive to treat obesity. Primary prevention is when the disease is absent and consists of promoting healthy eating, increasing physical activity, managing stress and improving well-being, avoiding excess weight gain during pregnancy, and controlling BMI in adolescents, as 80% of adolescents having obesity will stay obese into adulthood. PCPs also play an important role in preventing overmedicalization and the prevention of stigmatization [6-8]. Public health regulation is also crucial and needs to be addressed, as there are economic and environmental influences such as commercial promotion of foods high in fat and sugar [6].

Treatment in Primary Care

PCPs play a major role in obesity treatment as they provide the first contact to obesity patients [6]. Obesity is one of the most common chronic diseases that PCPs face. However, it is also an undertreated condition with many barriers to improving obesity care in the primary care setting. A cross-sectional study based on National Health and Nutrition Examination Survey (NHANES) data between 2011 and 2018 showed that only 40% of adults with overweight or obesity reported that they received counseling to lose weight [2]. Healthcare providers should ensure that they ask permission from their patients before discussing their weight or taking their anthropometric measurements [9]. The treatment of obesity includes lifestyle and behavioral therapy, pharmacotherapy, and bariatric surgery.

Counseling

Counseling is an essential component of managing obesity and should be done by using motivational interviewing (MI). In MI, inadequate terminology or judgmental language should be avoided, which may negatively affect patients’ feelings. PCPs should focus on positive behavioral changes, such as counseling on eating healthy food and increasing physical activity, and not focus on weight exclusively. Motivation is required to sustain long-term behavioral changes. There should also be management of the psychological effects of negative body image on the patients' self-esteem and assessment of eating disorders, depression, anxiety, stress, and sleep quality [6]. Along with MI and physical activity, it is important to set SMART (Specific, Measurable, Attainable, Realistic, and Time-based) goals with the patients [10]. SMART goals should take into consideration the patients' eating patterns, hunger cues, snacking habits, portion control, and physical activity [10]. Waist circumference should be measured as it is a predictor of visceral fat and cardiometabolic diseases, the cutoff being >88 cm in women and >102 cm in men. A protective profile against cardiometabolic risk is a gynoid morphotype, which is a low waist/hip ratio of <0.8 for women and <0.9 for men [6,11,12]. Waist circumference is also important to measure as it indicates obesity more accurately than BMI in elderly people, as older adults, despite having excess adipose tissue, have decreased muscle mass (sarcopenia) and loss of bone density [10,13]. Patients’ neck circumference should also be measured by the physicians as an increased circumference is present in sleep apnea, which is associated with obesity [10,14]. Patients should also be evaluated for comorbidities such as hypertension, type 2 diabetes, cardiovascular disease, asthma, sleep apnea, NASH (non-alcoholic steatohepatitis), gallbladder disease, colorectal cancer, kidney cancer, osteoarthritis, and breast cancer [6].

Lifestyle Interventions

Lifestyle interventions are important even if surgery or pharmacotherapy is advised. The United States Preventive Services Task Force (USPSTF) advises that patients with a BMI of over or equal to 30 undergo intensive multicomponent behavioral intervention. This comprises behavioral interventions like self-monitoring, goal-setting, stimulus control (where accessibility of tempting foods is reduced), stress control, improving sleep, and psychological therapy. USPSTF recognizes intensive intervention containing core and maintenance phases as at least 12 sessions of weight management counseling over a year and should last at least 1-2 years, with a 3-12-month phase of major weight loss, which is the core phase, followed by a 9-12-month maintenance phase. The multicomponent of this intervention is a combination of behavioral changes along with cognitive therapy, group sessions, and dietary education [2].

Reducing calorie consumption is recommended for weight loss. The American Heart Association/The Obesity Society/American College of Cardiology (AHA/TOS/ACC) promote reducing calories in diet by prescribing 1,500-1,800 kcal/day for men and 1,200-1,500 kcal/day for women and by cutting out on snacks creating a 500-750 kcal/day energy deficit, and also by recommending the Dietary Approaches to Stop Hypertension (DASH), Mediterranean, and low-carbohydrate diets, which are evidence-based diets that help in creating energy deficit by restricting certain food types [2,13]. The Mediterranean diet comprises legumes, vegetables, fruits, omega-3 fatty acids (like fatty fish, nuts, and cheese), and complex starches [6]. Processed and sugary foods and beverages should be limited or avoided. Fasting helps with positive metabolic adaptation by restricting calories [10]. Patients can join commercial programs (e.g., Weight Watchers and Jenny Craig), which have shown clinically significant effectiveness [10].

Physical activity is important for weight loss and maintenance. Moderate-intensity physical activity comprising ≥150 min/week, along with reduced calorie intake, leads to weight loss. Aerobic activities have the highest probability of reducing visceral adipose tissue. Resistance training maintains lean muscle, especially in post-menopausal women [2,13,15]. Regular physical activity also promotes visceral fat loss and reduces the risk of comorbidities [6]. Core training, which focuses on the exercise of abdominal and back muscles, can help improve posture, balance, and stability, and help with an increase in functional mobility [10]. Patients can maintain a diary to record their weight, food intake, physical activity, and sleep habits, which can help physicians work on modifiable behaviors [10].

Psychological Treatment

Psychological factors are very important in obesity management. Some patients may have eating disorders like binge eating, multiple snacking, and night eating syndrome, and these can be addressed by a psychologist, psychiatrist, and obesity specialists using cognitive behavioral therapy [6]. Patients having eating disorders have low self-esteem, and work needs to be done on building their self-confidence and improving body image using individual or group therapy [6]. Metformin and cognitive behavioral therapy should be considered to prevent weight gain in patients with severe mental illness who are treated with antipsychotic medications associated with weight gain [9].

Pharmacotherapy

Current weight loss medications used by PCPs are described in Table 1. PCPs should first ensure that none of the current medications the patients are on is causing weight gain. PCPs can refer to the Obesity Action Coalition (OAC), which has a list of drugs to be considered for replacement with other weight-neutral or weight-loss medications. Even though lifestyle modifications are important in obesity management, there is less success when used alone than in combination due to the obesity pathophysiology, where there are metabolic adaptations and changes in satiety and hunger signals, which lead to a regain in weight. Medications like glucagon-like peptide 1 (GLP-1)-receptor agonists (GLP-1RAs) are also beneficial for the comorbidities associated with obesity, like cardiovascular diseases. Antiobesity medications are indicated when the BMI is more than 30 kg/m^2^ or ≥27 kg/m^2^ with obesity-related comorbidities. They are also indicated when weight loss is not ≥5% of total body weight after 3-6 months with lifestyle interventions alone. Medications should be reassessed every three months if there is no significant weight loss. Food and Drug Administration (FDA)-approved medications are orlistat, phentermine, naltrexone, and bupropion. The GLP-1 approved for the treatment of obesity are liraglutide 3 mg daily and semaglutide 2.4 mg weekly. Tirzepatide is a GLP-1 agonist combined with a glucose-dependent insulinotropic polypeptide, which was approved for weight management by the U.S. FDA in December 2023 [10]. There are off-label medications also used for weight loss in obesity, such as metformin and topiramate.

**Table 1:** Weight loss medications used in primary care

Class of medication	Medication	Dosing/formulation and titration	Contraindications	Monitoring/cautions	Potential weight loss
Glucagon-like peptide-1 receptor agonists	Liraglutide (Saxenda)	0.6 mg initial dose for one week followed by dose escalation each week: 0.6, 1.2, 1.8, 2.4, and 3 mg	Family or personal history of medullary thyroid cancer or multiple endocrine neoplasia type 2; chronic pancreatitis. Contraindicated with sulfonylureas	Patient already on insulin should monitor for hypoglycemia and must be given clear and direct guidelines on how to titrate their insulin down accordingly	7% to 8% of body weight
	Semaglutide (Wegovy)	Subcutaneous injection: 0.25 mg weekly for 4 weeks, then titrating up to 0.5, 1.0, 1.7, and 2.4 mg for 4 weeks each; dose can be adjusted to the individual patient according to tolerance	Same as above	Same as above	15% to 16% of body weight
Glucose-dependent insulinotropic polypeptide and glucagon-like peptide-1 receptor agonist	Tirzepatide (Zepbound)	The recommended starting dosage is 2.5 mg injected subcutaneously once weekly. Increase the dosage in 2.5 mg increments after at least 4 weeks on the current dose. The recommended maintenance dosages are 5, 10, or 15 mg injected subcutaneously once weekly. The maximum dosage is 15 mg	Same as above; also serious hypersensitivity to tirzepatide or any of the excipients in tirzepatide	Same as above; also, use has been associated with gastrointestinal adverse reactions. Monitor renal function in patients with volume depletion. Monitor for depression or suicidal thoughts. Discontinue Zepbound if symptoms develop	22% to 23% of body weight
Lipase inhibitor; impairs digestion of dietary fat	Orlistat	60 mg with each fat-containing meal; do not take more than 3 capsules daily	Gastrointestinal side effects mainly with bowel movements and impaired absorption of fat-soluble vitamins	Consider checking levels of fat-soluble vitamins. Take a multivitamin daily. Monitor renal function in patients at risk of renal insufficiency	5% of body weight
Opioid antagonist/aminoketone antidepressant	Bupropion/naltrexone (Contrave)	Tablets: 90 mg bupropion/8 mg naltrexone. Week 1: one tablet in the morning. Week 2: one tablet twice daily. Week 3: two tablets in the morning and one tablet in the evening. Week 4+: two tablets twice daily	Nausea, constipation, headache, vomiting, dizziness, insomnia, dry mouth, and diarrhea. Increase in blood pressure (BP) and heart rate (HR). Hepatotoxicity: naltrexone exposure can cause hepatitis and liver dysfunction	Monitor for depression or suicidal thoughts and discontinue if symptoms develop. Monitor BP and heart rate, especially in those with cardiac or cerebrovascular disease	5% to 6% of body weight
Sympathomimetic; schedule IV stimulant	Phentermine	Phentermine HCl, 8, 15, or 30 mg orally in the morning; start low dose and titrate up as tolerated	Headache, hypertension, rapid or irregular HR, tremor, overstimulation	Check BP and HR on a regular basis; monitor on a monthly basis during dose titration	5% to 12% of body weight
Sympathomimetic; schedule IV stimulant/antiepileptic	Phentermine/topiramate (Qsymia)	Starting dosage: 3.75 mg phentermine/23 mg topiramate per day; at 14-day intervals, increase as tolerated; recommended dosage: 7.5 mg/46 mg per day; maximum dosage: 15 mg/92 mg per day; taper down the dose to prevent seizures when planning to discontinue	Paresthesia, dizziness, dysgeusia, insomnia, constipation, and dry mouth		10% of body weight

Orlistat, phentermine/topiramate (Qsymia), or phentermine alone have the lowest out-of-pocket prices in the United States [10,16]. However, it is important to give fat-soluble vitamins while on orlistat due to its effects on absorption (decreases absorption of dietary fat). Bupropion/naltrexone (Contrave) reduces hunger by stimulating the brain's reward or satiety center. Contrave should not be used in patients with seizure disorders or uncontrolled hypertension, patients on opioid therapy, or patients with bulimia [10]. The GLP-1 receptor agonists like liraglutide and semaglutide work by stimulating the pancreas to release insulin by stimulating GLP-1 receptors in the brain and inducing satiety. They also slow gastric emptying [10].

Bariatric Surgery

Surgery is recommended for patients with a BMI ≥ 40 kg/m^2^ or a BMI ≥ 35 kg/m^2^ with obesity-related comorbidities such as diabetes, hyperlipidemia, or hypertension. Bariatric surgery referral should be done in those patients who meet the criteria [17]. The expected weight loss with laparoscopic sleeve gastrectomy (LSG) is 20%-30%, and that for Roux-en-Y gastric bypass (RYGB) is 25%-35%. In uncontrolled type 2 diabetes patients, bariatric surgery should be considered as it is associated with greater weight loss, decreased number of glucose-lowering medications, and higher quality of life in surgical patients as compared to individuals on intensive diabetes medical therapy at three and five years after surgery [2,18]. A 2020 meta-analysis found that bariatric surgery was associated with a reduced risk of developing obesity-related conditions and reduced mortality [10,19]. Endoscopic procedures like intragastric balloons and sleeve gastroplasty are safer and minimally invasive procedures; however, they have minimal insurance coverage due to which it is of limited usefulness [2]. Patients after a bariatric procedure require lifelong medical follow-up and management after the procedure, as nutritional deficiencies arise depending on the type of bariatric surgery, which requires informed consent before surgery. Nutritional deficiencies of proteins, vitamins, minerals, and trace elements occur and need to be replaced by long-term mineral and multivitamin supplements [6]. Patients discharged from the bariatric surgical center should be annually reviewed by the PCPs for weight, nutritional intake, adherence to multivitamins and mineral supplements, and assessment for comorbidities, and should be treated for the nutritional deficiencies arising due to the surgery [9]. Some of the contraindications to bariatric surgery are inflammatory bowel disease, gastric ulcer, gastrointestinal motility disorders, current pregnancy, alcohol or substance abuse disorder, eating disorders, uncontrolled depression, psychosis, and inability to engage in lifestyle changes [20].

Scope for Improvement in Primary Care

In primary care, the barriers to addressing body weight are a lack of training and time [21,22]. According to one study, while 94% of internal medicine residents agreed that they should provide nutritional counseling, only 14% felt adequately trained to provide it [23]. PCPs often have time constraints, and it is difficult to make time to communicate and monitor patients about their weight management progress. For improvement, it is imperative for physicians to devote time to reviewing behavioral changes and lifestyle modifications and monitoring patient progress in order to prescribe medications effectively [2].

There is also a stigma associated with obesity. Most Americans (59%) believe that a lack of motivation or willpower is the cause of obesity, which is not true, as it is multifactorial due to genetic and environmental influences [24]. Patients also feel stigmatized to get treatment for obesity, and hence, it is important to use neutral terms and create a welcoming and non-judgmental environment where the patients can feel comfortable to discuss about weight loss measures. The Strategies to Overcome and Prevent (STOP) Obesity Alliance recommends all staff education and training to avoid bias and stigma. The STOP Obesity Alliance guide also recommends the 5A approach (assess, advise, agree, assist, and arrange) and includes the sixth A, “ask” for weight management counseling in primary care. The 5As framework has been shown to help keep patients motivated to lose weight, and patients of physicians who use the 5As framework lose more weight at 12 months [17,25]. Counseling should include shared decision-making (SDM) so that physicians and patients mutually agree on the treatment plan for obesity and can discuss risks, benefits, and treatment expectations [26]. Another barrier is poor reimbursement by health insurance for obesity counseling and antiobesity medications [20,22]. According to a national web-based survey of healthcare professionals, 53% of providers mentioned that improving reimbursement would help them counsel patients about obesity better [22]. Expanding health access for patients with telehealth can also improve management in primary care. With the help of telehealth visits, monitoring behavioral changes and progress in weight loss and monitoring medications can be achieved. Team-based care consisting of dietitians, nurses, and behavioral therapists can help achieve lifestyle modifications for weight loss.

## Conclusions

This article covered the comprehensive management of obesity and went through various aspects of primary, secondary, and tertiary prevention of obesity. Primary prevention is important as obesity, once established, is difficult and expensive to treat. This article also covered barriers associated with obesity management and suggested various strategies to improve the current management of obesity. We discussed the importance of counseling and MI to bring about positive long-term behavioral changes to lose weight and the treatment for obesity. The article aimed to examine the scope for improvement by expanding healthcare access with the help of telehealth and team-based care. A multidisciplinary team comprising PCPs, nurses, dietitians, psychiatrists, and physical activity specialists is needed to treat obesity effectively. Public health regulations need to bring changes due to environmental and economic influences, like foods high in fat and sugar, and their commercial promotion. Future research can study cost-effectiveness and ways to improve obesity care in primary care.
